# Supplementary material for: Pathogenicity and transmission of Morganella morganii in honey bees
Source: PLoS Pathog. 2025 Oct 22;21(10):e1013613. doi: 10.1371/journal.ppat.1013613 (PMC12543119; doi:10.1371/journal.ppat.1013613)
Supplement: S1 Appendix — Fig A. Heatmap of Average Nucleotide Identity of Morganella strains. The one isolated from the Varroa mite shows an average identity of 92%. The genome assembled in this study is highlighted. Fig B. The transmission of M. morganii through social contacts. We injected the engineered M. morganii:pBTK520 into bees and co-housed these bees with naïve ones. The engineered M. morganii:pBTK520 developed high titers in the injection group, which is absent in bees in the co-housing group and PBS group. The data suggest that the symbiont can’t be transmitted through social contacts. Table A. The bacterial species isolated from mites. All mites were rinsed to remove microbes from the body surface. The mites were then homogenized to isolate single colonies under various conditions. In total, 33 bacterial species were isolated. Morganella is particularly interesting because it has been reported in both bees and mites in a previous metagenome study, suggesting a mutual transmission. Table B. The genome assembly statistics of M. morganii CYJ1 genome. The genome is assembled into a single contig, with 3.8 Mbp. Out of 124 conserved single-copy genes in the bacterial kingdom, 122 were identified, and the remaining 2 were fragmented. Table C. Genomes of Morganella morganii for ANI (Average Nucleotide Identity) analysis. We randomly selected 20 complete genomes to perform ANI and AAI comparisons with the strain CYJ1. (DOCX) [file ppat.1013613.s001.docx]

**Supplementary Material for**

Pathogenicity and transmission of *Morganella* *morganii* in honey bees

Yijun Chen^1^ and Qiang Huang^1*^

^1.^ Honeybee Research Institute, Jiangxi Agricultural University, Zhimin Ave. 1101, Nanchang, 330045, China.

* [qiang-huang@live.com](mailto:qiang-huang@live.com) (QH);

Running title: *Morganella* in honey bees

**Supplementary method**

*M. morganii* CYJ01 engineering

The *Morganella morganii* CYJ01 was cultivated on an LB plate. To prepare electrocompetent cells, a single colony of *M. morganii* CYJ01 was added to 50 mL of LB and incubated at 35°C for 12 hours. Then the liquid media was centrifuged at 7,000 rpm for 10 min to harvest the pellet. The pellet was washed with autoclaved ultra-pure water three times, and the pellet was re-suspended in 1 mL of autoclaved 10% glycerol. For the electroporation, 100 µL electrocompetent *M. morganii* CYJ01 was mixed with 500 ng pBTK520 (Addgene #110604) and incubated on ice for 20 min. The electroporation was conducted using Bio-Rad (GenePulser Xcell), with the following parameter: voltage 1800, capacitance 25, resistance 1, and cuvette 1mm. After the electroporation, cells were transferred into 50 mL LB and incubated at 35°C overnight. The electroporated *M. morganii* CYJ01 cells (50 µL) were then plated on LB and Spectinomycin (60µg/mL) for 12 h.

**Supplementary Results**

**Table A.** The bacterial species isolated from mites. All mites were rinsed to remove microbes from the body surface. The mites were then homogenized to isolate single colonies under various conditions. In total, 33 bacterial species were isolated. *Morganella* is particularly interesting because it has been reported in both bees and mites in a previous metagenome study, suggesting a mutual transmission.

|  | **Inferred bacterial species from isolated single colonies using 16S sequencing** |
| --- | --- |
| 1 | >KT029661.1 Uncultured bacterium clone 5-9 16S ribosomal RNA gene *Morganella* |
| 2 | >MT796126.1 Moniliella sp. isolate CK-Bc1 small subunit ribosomal RNA gene, partial sequence; internal transcribed spacer 1, 5.8S ribosomal RNA gene, and internal transcribed spacer 2, complete sequence; and large subunit ribosomal RNA gene, partial sequence |
| 3 | >CP046394.1 Bombella sp. ESL0368 chromosome, complete genome |
| 4 | >KT983982.1 Bacillus circulans strain HMF2507 16S ribosomal RNA gene, partial sequence |
| 5 | >MT544719.1 Staphylococcus hominis subsp. novobiosepticus strain 4149 16S ribosomal RNA gene, partial sequence |
| 6 | >MZ276284.1 Microbacterium esteraromaticum strain DSM 8609 16S ribosomal RNA gene, partial sequence |
| 7 | >HM449702.1 Micrococcus luteus strain PCSB6 16S ribosomal RNA gene, partial sequence |
| 8 | >AY370192.1 Candidatus Gilliamella apicola clone pAJ206 16S ribosomal RNA gene, partial sequence |
| 9 | >FJ217185.1 Micrococcus sp. BBAPs-01d 16S ribosomal RNA gene, partial sequence |
| 10 | >KC887933.1 Proteobacterium K14 16S ribosomal RNA gene, partial sequence |
| 11 | >KM464072.1:10-1434 Uncultured bacterium clone 11_Ne_15 16S ribosomal RNA gene |
| 12 | >KR780376.1 Staphylococcus sp. CS21 16S ribosomal RNA gene, partial sequence |
| 13 | >KT029729.1:9-1431 Uncultured bacterium clone 9-14 16S ribosomal RNA gene |
| 14 | >MF399389.1 Paenibacillus urinalis strain 38 16S ribosomal RNA gene, partial sequence |
| 15 | >MH518227.1 Staphylococcus hominis strain CLC-M5 16S ribosomal RNA gene, partial sequence |
| 16 | >MH665979.1:32-1444 Micrococcus luteus strain FC1737 16S ribosomal RNA gene |
| 17 | >MN746191.1:2-1454 Bacillus cereus strain LXJ76 16S ribosomal RNA gene |
| 18 | >MT023404.1 Staphylococcus caprae strain XM28 16S ribosomal RNA gene, partial sequence |
| 19 | >MT197286.1 Staphylococcus capitis strain IGM6-16 16S ribosomal RNA gene, partial sequence |
| 20 | >MT225639.1 Roseomonas mucosa strain 1910ICU262 16S ribosomal RNA gene, partial sequence |
| 21 | >MT367816.1:4-1420 Pantoea eucrina strain OsEp_Plm_15P14 16S ribosomal RNA gene |
| 22 | >MW433652.1 Agrococcus jenensis strain SCR56 16S ribosomal RNA gene, partial sequence |
| 23 | >MZ734327.1 Micrococcus luteus strain PP.K.15 16S ribosomal RNA gene, partial sequence |
| 24 | >NR_181622.1 Bifidobacterium apousia strain W8102 16S ribosomal RNA, partial sequence |
| 25 | >OK135640.1 Pseudomonas sp. strain YL-203 16S ribosomal RNA gene, partial sequence |
| 26 | >OK136213.1:5-1353 Brevundimonas intermedia strain YL-124 16S ribosomal RNA gene |
| 27 | >OK147814.1 Stutzerimonas kunmingensis strain YL-233 16S ribosomal RNA gene, partial sequence |
| 28 | >ON045811.1:4-1452 Bacillus cereus strain NS25 16S ribosomal RNA gene |
| 29 | >ON795198.1:9-1455 Bacillus cereus strain SA275C1 16S ribosomal RNA gene |
| 30 | >ON799124.1 Priestia sp. strain R2A P SY-1 16S ribosomal RNA gene, partial sequence |
| 31 | >ON926587.1 Staphylococcus hominis subsp. novobiosepticus strain R14 16S ribosomal RNA gene, partial sequence |
| 32 | >OP862446.1 Staphylococcus equorum strain NR_5-1 16S ribosomal RNA gene, partial sequence |
| 33 | >OQ405510.1 Cytobacillus kochii strain C41 16S ribosomal RNA gene, partial sequence |

**Table B.** The genome assembly statistics of *M. morganii* CYJ1 genome. The genome is assembled into a single contig, with 3.8 Mbp. Out of 124 conserved single-copy genes in the bacterial kingdom, 122 were identified, and the remaining 2 were fragmented.

| Genome size | 3.8 Mb |
| --- | --- |
| Total ungapped length | 3.8 Mb |
| Number of chromosomes | 1 |
| Number of scaffolds | 1 |
| Scaffold N50 | 3.8 Mb |
| Scaffold L50 | 1 |
| Number of contigs | 1 |
| Contig N50 | 3.8 Mb |
| Contig L50 | 1 |
| GC percent | 50 |
| Genome coverage | 1000.0× |
| Assembly level | Complete Genome |
| Genes | 3,720 |
| Protein-coding | 3,531 |

**Table C.** Genomes of *Morganella morganii* CYJ01 for ANI (Average Nucleotide Identity) analysis. We randomly selected 20 complete genomes to perform ANI and AAI comparisons with the strain CYJ1.

| **Strian/Isolate** | **Assembly** | **Genome size** | **ANI** | **Host** |
| --- | --- | --- | --- | --- |
| CYJ1 | GCA_044772875.1 | 3.8 Mb | NA | mite |
| Morganii KT | GCF_000286435.2 | 3.8 Mb | 91.9% | human |
| UM869 | GCA_025398975.1 | 3.8 Mb | 91.9% | human |
| FDAARGOS_63 | GCF_000783955.2 | 4.0 Mb | 92.0% | human |
| FDAARGOS_172 | GCA_001558895.2 | 3.9 Mb | 91.9% | human |
| KC-Tt-01 | GCA_002891475.1 | 3.8 Mb | 92.1% | human |
| subsp. morganii | GCF_006094455.1 | 3.9 Mb | 91.9% | ATCC Missing |
| Jiangxi | GCA_013378135.1 | 3.8 Mb | 91.9% | loach_fish |
| GDMM86 | GCF_016618235.1 | 4.1 Mb | 91.8% | environment |
| Colony326 | GCF_016925135.1 | 4.1 Mb | 92.0% | food |
| Colony180 | GCF_018798965.1 | 4.1 Mb | 92.0% | food |
| FAM24685 | GCF_019242835.1 | 4.2 Mb | 91.0% | cheese |
| FAM24679 | GCF_019242955.1 | 4.1 Mb | 91.1% | cheese |
| MMAS2018 | GCF_020790175.1 | 4.0 Mb | 92.0% | pig |
| S164-3 | GCF_022369415.1 | 4.0 Mb | 91.9% | Manis javanica (reptile) |
| Sample-M-2023 | GCF_030127185.1 | 3.9 Mb | 91.9% | snake |
| SDTA-1 | GCF_031851815.1 | 4.0 Mb | 92.0% | cattle |
| 41 | GCF_038406725.1 | 4.0 Mb | 91.8% | fish |
| M-D1C1-1 | GCF_044161685.1 | 4.3 Mb | 91.1% | Mink (mammal) |
| M-D1C1-2 | GCF_044161715.1 | 4.3 Mb | 91.1% | Mink |
| HIS2824 | GCF_949789215.1 | 4.2 Mb | 91.2% | fish |
| 23 | GCF_038406735.1 | 3.9 Mb | 91.8% | fish |
| OT11 | GCF_041319835.1 | 4.3 Mb | 92.0% | frog |
| MP63 | GCF_010748915.1 | 4.0 Mb | 92.0% | Wast water |
| DZ1 | GCF_037482145.1 | 4.3 Mb | 91.1% | water |
| 2431 | GCA_015892125.1 | 4.4 Mb | 91.4% | fly larvae |
| 2232 | GCA_015892085.1 | 4.4 Mb | 91.2% | Air of fly rearing |


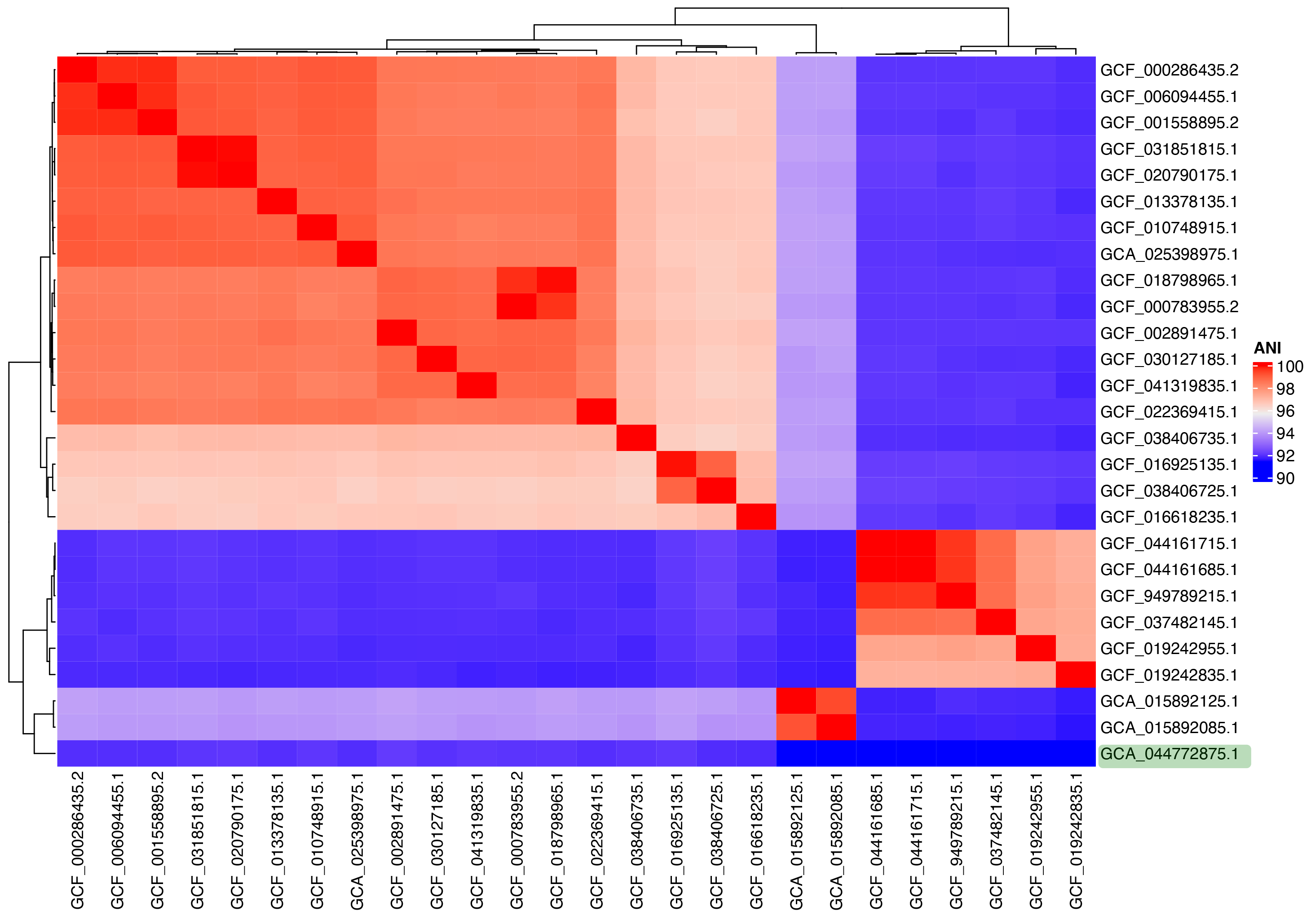


Fig A. Heatmap of Average Nucleotide Identity of *Morganella* strains. The one isolated from the *Varroa* mite shows an average identity of 92%. The genome assembled in this study is highlighted.

Fig B. The transmission of *M. morganii CYJ01* through social contacts. We injected the engineered *M. morganii*:pBTK520 into bees and co-housed these bees with naïve ones. The engineered *M. morganii*:pBTK520 developed high titers in the injection group, which is absent in bees in the co-housing group and PBS group. The data suggest that the symbiont can’t be transmitted through social contacts.
