# Supplementary material for: Pathogenicity and transmission of Morganella morganii in honey bees
Source: PLoS Pathog. 2025 Oct 22;21(10):e1013613. doi: 10.1371/journal.ppat.1013613 (PMC12543119; doi:10.1371/journal.ppat.1013613)
Supplement: Attachment — Submitted filename: Response_to_Reviewers.docx [file ppat.1013613.s004.docx]

Dear Editor,

We appreciate the editor's and reviewers’ comments, which substantially improve the clarity of the manuscript. All comments have been replied to point by point, and the corrections are highlighted in color.

\# Editor’s comments:

Major consistent points were raised by all three reviewers with the writing, particularly details of the experimental protocols, the bacterial strain origin, and citations of the literature to emphasize the importance of the work. One reviewer raised concerns regarding the use of gnotobiotic bees. While we do not expect repetition of all the work in wild bees with full microbiota, this is a flaw that should be clearly acknowledged as important for future studies.

**Response:** We have clarified the experimental design details below. We have specifically stated that “This study is limited to the germfree bees, and it is interesting to investigate the impacts of gut microbes on *M. morganii* for future studies” (Line: 219-220).

**Reviewers' Comments:**

**Part II – Major Issues: Key Experiments Required for Acceptance**

Reviewer #1: The bacteria investigated is a particular strain of M. morganii that you claim to have isolated in [17] (M. morganii CIJ01), as stated in L51; nonetheless, no reference to this pathogen is provided in [17]: is this reference correct? Make sure to provide a complete explanation as to the source of the organisms you have used.

**Response:** Sorry for the confusion. *M. morganii* is an uncultured bacterium in honey bees and mites, previously inferred from a sequencing study. We have clarified that “In this study, we isolated the first strain of *Morganella morganii* in *Varroa* mites, which is highly virulent in honey bees” (Line: 56-57).

L83: I think that the last sentence is incomplete, as I suspect that you not only calculated the number of cells in the 10-7 inoculum, but also used this titer to obtain the 10-5 and 10-3 solutions as well. If so, I’d reformulate as “To estimate the number of CFUs in each dilution, we first counted the number of cells in 1µL of 10-7 inoculum (N=11 replicates); then, we derived the titer of the 10-5 and 10-3 dilution, as a proportion of the average number of CFUs counts in the 10-7 dilution.”. Also, I think this sentence would be better placed in L77, before you describe how you used the inoculum.

**Response:** We have reformatted and replaced the statement as: “To estimate the number of CFUs in each dilution, we first counted the number of cells in 1µL of 10^-7^ inoculum (N=11 replicates); then, we derived the titer of the 10^-5^ and 10^-3^ dilutions, as a proportion of the average number of CFU counts in the 10^-7^ dilution. We injected 1μL inoculum of 10^-3^ (N=127 pupae), 10^-5^ (N=126 pupae), and 10^-7^ (N=128 pupae) into the pupae” (Line: 87-90).

L167-170: Clarify how you get to 92.1%: from Figure 3, I suspect that you divided 35 infected mites by 38 (those that were transferred on infected bees): however, none of these numbers appear in the main text. The sample size (38) and the formula should be stated in Methods, and the outcome (35 out of 38) in the Results. Furthermore: unless you acknowledge the basic reproductive number (R0) of Morganella, the use of the terminology “transmission rate” is improper (as the transmission rate is R0 over time): I suggest you to refer to “cumulative incidence over X days/hours” or “attack rate over X days/hours” (See caption of Figure 3, too)

**Response:** We have clarified in the material and methods section that “We homogenized the mite for CFU counting to infer the proportion of mites that acquired the fluorescent *M. morganii* (N=38 mites) from the injected bees” (Line: 115-116), also in the results section that “In total, 35 out of 38 mites harbored *M. morganii* once they parasitized bees that had been injected with *M. morganii*. Thus, 92.1% of mites acquired *M. morganii* from infected bees through parasitism.” (Line: 172-174).

We have revised the Figure 3 caption and legend as “Figure 3. Acquisition of *M. morganii* from bees and mites. We found 35 out of 38 mites that were transferred to bees injected with *M. morganii*, harbored *M. morganii* (A). Thus, the cumulative incidence of *Varroa* mites’ acquiring *M. morganii* from bees is 92.1% in 24 hours through parasitism. The mites that carry *M. morganii* vector the pathogenic bacteria to other naïve bees. The cumulative incidence of bees’ acquiring *M. morganii* from mites is 68.4% (B). This pathogenic bacterium resulted in a 72% mortality rate among recipient bees, which is comparable to the 62.4% mortality rate in the injection group (C).” (Line: 359-364).

Reviewer #2: While the experimental work is strong, the manuscript suffers from weak writing and poor structure. The narrative would benefit from clearer organization, more precise framing of the research questions, and stronger transitions between sections.

The most important is that the manuscript lacks a Data Availability Statement. There also seems to be no raw data sets, which nowadays is a standard. If I somehow missed this, then the authors and the editors should ignore this comment.

**Response:** The Data Availability Statement is available in the submission system. We have now included it in the main text as “All study data are included in the article and supplementary material. The genome and gene annotation in deposited in NCBI GCA_044772875.1. The data used to generate the figures in the main text and supplementary material are provided in data_deposit.xlsx.” (Line: 227-229).

The introduction is very brief and does not adequately place the work in the context of existing literature. I am missing clear questions and expectations. It should be expanded, putting the question in a broader frame
**Response:** We have clarified the research question as “In a previous study, a few bacterial pathogens were inferred in mites and honey bees using 16S sequencing [16]. We hypothesize that the *Varroa* mite transfers and acquires bacterial pathogens upon feeding the bees. To investigate the virulence and transmission routes of the bacteria vectored by *V. destructor*, we first isolated and cultured those bacteria. Then, we injected the bacterial inoculum into bees to observe the bacterial proliferation and bee mortality. Specifically, we isolated the first strain of *Morganella morganii* in *Varroa* mites, which is highly virulent in honey bees” (Line: 52-57).

The manuscript cites only 37 references, which is limited for a study of this scope. The authors should broaden the reference list to include relevant recent work and ensure that the findings are well contextualized in the current literature.

**Response:** There are only a limited number of studies on the bacteria vectored by *V. destructor*. Hubert et al (2015) detected bacteria that might be carried by mites from beehive debris [15]. Hubert et al (2016) inferred a few bacteria in mites and bees using 16S sequencing, indicating mutual transmission [16]. None of the above studies attempted to isolate and culture the bacteria. The most exciting paper so far is Zhang et al (2013), who isolated the first *Enterococcus* bacterium in bees*,* and *Varroa* mites vector this bacterium [17]. The negative impact of pathogenic bacteria vectored by *Varroa* has been rarely studied. This field will attract more research interest, and the number of related literature will expand. Deformed wing virus is the best studied virus vectored by *V. destructor*. Its transmission route and the exact dispersal rate have been deciphered recently by Lamas et al (2024) [14]. We attempted to gather as much relevant literature as possible. If we have missed any critical or relevant literature, we are happy to include it.

The discussion is very basic and does not sufficiently explore the implications of the results, limitations of the study, or how findings relate to broader knowledge in the field. Authors should expand this section to highlight significance, compare results with similar studies, acknowledge limitations, and suggest directions for future research.

**Response:** We have clarified the highlight as “In this study, we isolated the first *M. morganii* in *Varroa* mites and highlighted that this pathogenic bacterium is highly virulent in honey bees.” (Line: 191-192), “In our study, *M. morganii* is transmitted through the feeding of *Varroa* mites.” (Line: 203-204).

We have compared those highlights with other studies as “However, the virulence of *M. morganii* is rarely investigated. A previous study found that 10^5^ CFU of *M. morganii* resulted in 100% mortality in flies, and a lower dosage of 10^2^ CFU caused 44% mortality [32]. *M. morganii* also reduced the flight ability and pupal weight of the flies [33]. In nematodes, 10^6^ CFU of *M. morganii* leads to 100% mortality of infected nematodes in 24 hours, and 10^2^ CFU leads to 20% mortality [27]. In our data, *M. morganii* caused over 30% mortality in honey bees at a low dosage of 10^2^ CFU. However, the titer of *M. morganii* increases to 10^10^ CFU within 24 hours, thereby enhancing the case fatality rate, leading to the observed acute mortality.” (Line: 193-200).

We have acknowledged the limitation and suggested the future direction as “This study is limited to the germfree bees, and it is interesting to investigate the impacts of gut microbes on *M. morganii* for future studies” (Line: 219-220).

Line 64 – 16s sequencing is not clearly described what has been done. Also, where are those sequences?

Are they deposited somewhere?

**Response:** 16S sequence is not uploaded because we have already assembled its genomes and gene annotation under NCBI genome GCA_044772875.1. The raw HiFi reads are uploaded under this genome assembly in NCBI as well. We have revised the statement as “We sequenced a single colony of *M. morganii*, assembled and annotated the genome under the NCBI genome GCA_044772875.1 (Figure 1A-B)” (Line: 131-132).

Line 68-67 – where is the genome annotation?

**Response:** The genome annotation is under the NCBI genome GCA_044772875.1. Here is the link: https://ftp.ncbi.nlm.nih.gov/genomes/all/GCA/044/772/875/GCA_044772875.1_Mmor_CYJ1/

Line 119 – How was the genome assembled?

**Response:** We have clarified the procedure as “The DNA of a single colony of *M. morganii* was extracted using Sodium Dodecyl Sulfate (SDS) and sheared to 30 Kbp to be sequenced in PacBio Sequel II according to the Illumina DNA preparation instructions. The highly accurate long reads (HIFI) were assembled using Flye assembler (Version 0.3-3) with default parameters[19], and the gene features were annotated using the NCBI Prokaryotic Genome Annotation Pipeline[20]. To assess the completeness of the genome, we aligned the protein sequence to the conserved single-copy orthologs of the bacterial kingdom (BUSCO Bacteria_odb10 database)” (Line: 76-81). We have summarized the genome feature as “Its genome is 3.8 Mbp in a single contig, with 3,531 protein-coding genes (Table S2), including 122 complete and 2 fragmented BUSCOs.” (Line: 133-134).

Line 182 – why are the PBS bees also dying?

**Response:** We have explained as “The injection may cause physical damage to the bees, reflected by the mortality in the PBS group” (Line: 186-187).

Line 196 – citation?

**Response:** reference is added and the statement has been revised as “For example, another opportunistic pathogen of honey bees, *Serratia marcescens*, caused approximately 20% mortality when infected through feeding, while injecting it leads to 100% mortality [34].”(Line: 201-203).

Reviewer #3: 1. One of the drawbacks/concerns that I have with this work is that it has been conducted in adult bees without any microbiome (gnotobiotic). It has been shown over the last 10 years how important the microbiome of an arthropod (most organisms) is maintaining a sort of homeostasis and to keep possible pathogenic bacteria at a level that it does not impact the individuals health. This can take the form of antibacterial peptides that are bacteriostatic or other PAMPs which work in a similar manner. There Sia whole host of work done in mosquitoes and ticks displaying this. This would then make the set of experiments here, of displaying a pathogenic potential, very one-sided. I would strongly suggest the authors use their GFP generated isolate to conduct the experiments in regular bees from a natural/lab colony that has a regular microbiome to determine if what they suggest is valid in nature especially given their discussion section. The authors do not at all address in their discussion this missing aspect associated with the absence of a microbiota.

**Response:** This is a good suggestion, and we have planned the follow-up project investigating the impact of gut symbionts on *M. morganii*, as well as identifying the essential gene in *M. morganii* using the Tn mutagenesis library. In the manuscript, we have acknowledged the limitation as “This study is limited to the germfree bees, and it is interesting to investigate the impacts of gut microbes on *M. morganii* for future studies” (Line: 219-220).

2. My second concern is trying to understand how many mites were used for the acquisition studies and also how many where used for the transmission studies per bee. Also were all mites that fed on naive bees infected ? What criteria was used to determine the transmission rate if not all mites were infected at the time of feeding on naive bees?

**Response:** We have clarified that “First, we injected 25 gnotobiotic bees with the fluorescent *M. morganii*, transferring one mite to each bee’s body surface for 24 hours, providing 5 replicates of this experiment (N=125 infected bees and 125 mites): we refer to this as the injection group. At 24 hours, we homogenized the mite for CFU counting to infer the proportion of mites that acquired the fluorescent *M. morganii* (N=38 mites) from the injected bees. After 24 hours, we randomly selected 15 of those mites, transferring each one to a naïve bees’ body surface for 3 days (5 replicates, N = 75 naive bees and 75 infected mites) as the recipient group.” (Lines: 112-118). All live mites acquired *M. morganii* once they parasitized the *M. morganii* injected bees. Thus, we can calculate the proportion of bee acquire *M. morganii* from the mites. In the manuscript, we have clarified as: “Since all alive mites acquired *M. morganii* from the infected bees, the cumulative incidence of naïve bees acquiring *M. morganii* from the infected mites is 68.49%” (Line: 178-180).

3. Overall the conclusion is not very convincing (lines 209-213) , without such an experiment being done. Bee colonies can be kept at lower temperature and mites (infected with the GFP isolate) can be introduced along with some of the bees , from the same colony, to see if this is possible. This is only a suggestion for the future.

**Response:** We have acknowledged the limitation of using germfree bee as “This study is limited to the germfree bees, and it is interesting to investigate the impacts of gut microbes on *M. morganii* for future studies” (Line: 219-220).

**Part III – Minor Issues: Editorial and Data Presentation Modifications**

 Reviewer #1: The study focus on the mite Varroa destructor, but its name is only mentioned once in the manuscript body: instead, the generic “mites” is used throughout; I think it would be useful to remind it here and there, either by using its specific name, or by using “Varroa mites” instead of just “mites”. This should be done especially in Introduction and Discussion, when it is not clear whether you are talking of mites “in general”, or V. destructor. One example of this ambivalence is in L41: “The mite associated virus has been extensively investigated…”.

**Response:** We have replaced “mite” with “*Varroa* mites” throughout the text.

Throughout the Manuscript, M. morganii it is referred to either as to as “M. morganii CIJ01” or, once engineered for fluorescence, M. morganii:pBTK520. While I find correct to explicit the strain when specific results are presented, most of the instances require the binomial nomenclature only, which would improve readability (e.g. in L53: “transmission of M. morganii CIJ01 depends on mites”, where this sentence is not only applicable to this strain, but to the species altogether). Similarly, I also think that you can omit the code “pBTK520” throughout the Manuscript, after you specify the engineering of the mites in Methods: the reader knows that your mites are fluorescent, and you can still remind of it when necessary by specifying “fluorescent M. morganii”, “tagged M. morganii” or alike. This would improve readability, while losing no information and highlighting the scope of your work.

**Response:** We have deleted “CYJ01” and “pBTK520” and used “fluorescent *M. morganii*” throughout the text.

Following, I list a line-by-line revision of the main body of the text, including figures and supplementary material when appropriate.

L12: Replace with "bees body fat" or "bee’s fat body tissue". Also in L26, L36

**Response:** We have replaced “fat body of bees” with “bee’s fat body tissue” (Line” 12, 26, 36).

L12-14: Unclear: what virus? Is it more than one? Is it the virome?

**Response:** We have clarified as “The *Varroa* mite-associated deformed wing virus has been extensively studied because it can be found in each individual mite and causes bee mortality.” (Line: 12-13).

L17: Virulence is a measure of the harm caused by a pathogen to a host; here, you seem to refer to mortality: in this case, a more appropriate terminology would be “case fatality rate”

**Response:** We have revised as “This pathogenic bacterium exhibited a high case fatality rate” (Line: 17).

L19: For simplicity, I suggest to replace "...social contacts. Instead, the transmission of M. morganii depends on bees." with "...social contacts, while it can be transmitted from mites to bees, and vice versa.".

**Response:** It has been revised as suggested (Line: 19).

L20: The transmission rate is R0 over time, so it does not apply to this study. I therefore suggest to refer to “cumulative incidence over X days/hours” or “attack rate over X days/hours”.

**Response:** We have revised as “The cumulative incidence of transmitting *M. morganii* from infected bees to mites is 92.1%, and 68.49% from infected mites to naïve bees.” (Line: 19-21).

L21: As the present study do not analyse M. morganii-induced mortality in the field, I strongly suggest to replace “our data partially explain” with “our data aligns with”, or alike.

**Response:** We have replaced “our data partially explain” with “our data aligns with” (Line: 21).

L27: I think "Despite this", "However", "Nonetheless", "Yet" or similar would be more adequate than "Comparatively" here

**Response:** We have replaced “Comparatively” with “However” (Line: 27).

L28: As stated, it seems that mortality is transmitted to mite feeding, which is nonsense. I suggest rewriting as "A low dosage of M. morganii, which is transmitted through mite feeding, causes high mortality…"

**Response:** We have revised as “A low dosage of *M. morganii*, which is transmitted through mite feeding, causes high mortality in pupae and adult bees” (Line: 28-29).

L29: M. morganii was well known before of this study: I strongly suggest to replace "reveal a novel" with "deepens the knowledge about a", unravel the transmission dynamics of a", or alike

**Response:** We have revised it as “Our data deepens the knowledge about the bacterial pathogen, vectored by mites.” (Line: 29-30).

L38: I think this sentence could benefit from some rearrangement: as it is, it sounds like all mites transmit DWV and "a bunch of" other viruses, sounding somewhat casual. I suggest something like "Upon feeding, the mites can inject a variety of viruses into the haemolymph of bees: among them, the deformed wing virus (DWV) is one of the most occurring, causing developmental disorders in infected individuals."

**Response:** We have revised the statement as “Upon feeding, the mites can inject a variety of viruses into the hemolymph of bees: among them, the deformed wing virus (DWV) is one of the most prevalent, causing developmental disorders in infected individuals” (Line: 38-40).

L38: As you are talking of DWV only, and refer to a publication which has studied it in V. destructor only, rewrite as "DWV has been extensively investigated because of its ubiquity in Varroa destructor".

**Response:** It has been revised as suggested (Line: 42).

L42: I suggest rephrasing as "...bee, as validated by fluorescent tagging, and infected individuals serve as…"

**Response:** It has been revised as suggested (Line: 43).

L44: As this two sentences are directly linked, I suggest not to separate them with a full stop; instead, I suggest stating it as "...bacteria: a previous 16S…"

**Response:** It has been revised as suggested (Line: 45).

L45: See comment in L38

**Response:** We have revised the statement as “The *Varroa* mites also harbor several bacteria: A previous 16S sequencing study identified a few bacteria in mites and honey bees, including *Morganella*, *Spiroplasma*, and *Enterococcus*, suggesting these bacteria may transmit between them” (Line: 44-47).

L46: To avoid repetition, I suggest replacing "bees and mites" with "them"

**Response:** We have replaced "bees and mites" with "them" (Line: 47).

L46-48: This paragraph needs rephrasing. These are the main issues I find in it:

1. It is not clear whether these bacteria were never cultured previously by anyone, or if it was a limitation of [15] only.

2. 3 bacterial genera are specified (amongst which the subject of the Manuscript), and “a few others”: which bacteria are you referring to? Whose transmission route and virulence remain unclear?

3. It is not clear how the fact that “there isn’t a ubiquitous bacterium harboured by each individual mite” (which mite?) links with a lesser attention to mite-vectored bacteria.
Maybe, this paragraph could be replaced by something in the lines of “Given the difficulties in cultivating these pathogens, and the complexity of the bacteria community found in Varroa mites, this field of study remains largely unexplored.”? Also, see comment for L17

**Response:** We have revised the statement as “Given the difficulties in cultivating these pathogens and the complexity of the bacterial community found in *Varroa* mites, this field of study remains largely unexplored. Until recently, *Enterococcus faecalis* was isolated from dead bees, which is vectored by *Varroa* mites. *E. faecalis* caused substantial mortality in the honey bee *A. mellifera* [16]. Thus, the mite also serves as a vector for pathogenic bacteria of honey bees. However, the transmission routes of *E. faecalis* are not fully understood.” (Line: 47-51)

L49-50: This paragraph needs rephrasing. These are the main issues I find in it:
1. As stated, it seems that E. fecalis nowadays in not isolated from dead bees, while it is.
2. As stated, it seems that isolating E. fecalis caused mortality in (already dead) bees, which is unlikely.
3. It is unclear how the last sentence follows from these premises

**Response:** We have revised the statement as “Until recently, *Enterococcus faecalis* was isolated from dead bees, which is vectored by *Varroa* mites. *E. faecalis* caused substantial mortality in the honey bee *A. mellifera*” (Line: 48-50).

L52: If M. morganii CYJ01 is the strain that you have used, you cannot state that you isolated “a highly virulent strain of M. morganii CYJ01”. Maybe replace with “...a highly virulent strain of M. morganii (M. morganii CYJ01) in mites.”?

**Response:** We have revised the statement as “Specifically, we isolated the first strain of *Morganella morganii* in *Varroa* mites, which is highly virulent in honey bees.” (Line: 56-57).

L55: Given that you are commenting your results, move to “Discussion”. As the pathogen (M. morganii) was already known, “revealed” sounds off; I’d replace with something like “Our data revealed a high lethality induced by M. morganii through mite bite: this might partially explain bee colony losses during late autumn and winter, when intraspecific contact among bees is higher.”.

**Response:** We have revised it as suggested (Line: 59-61).

L61: Explain the acronym PBS (I suppose Phosphate Buffered Saline)

**Response:** We have revised the statement as “homogenized them in 500 μL Phosphate Buffered Saline (PBS)” (Line: 66-67).

L63: Which broth have you incubated “on regular incubators”, and which in CO2?

**Response:** We have clarified that “We prepared two plates for each culturing medium and incubated one plate in a CO_2_ incubator and the other in a regular incubator.” (Line: 69-70).

L65: Explain the acronym OD (I suppose Optical Density)

**Response:** We have revised the statement as “We recorded the Optical Density (OD) at 2-hour intervals” (Line: 73).

L66: Unclear: 12 replicates of which sample/passage?

**Response:** We have revised the statement as “N = 12 replicate samples for each concentration” (Line: 74).

L68: Specify “The DNA of a single colony of M. morganii...”

**Response:** We have specified “The DNA of a single colony of *M. morganii*” as suggested (Line: 76).

L68: Explain the acronym SDS and provide reference; L68: Explain the means of shearing DNA and provide reference of used instrumentation

**Response:** This is a standard DNA extraction protocol for Illumina sequencing. We have clarified as “The DNA of a single colony of *M. morganii* was extracted using Sodium Dodecyl Sulfate (SDS) and sheared to 30 Kbp to be sequenced in PacBio Sequel II according to the Illumina DNA preparation instructions” (Line: 76-77).

L69: Provide reference for PacBio instrumentation

**Response:** The PacBio machine is called Sequel II (Line: 77).

L69: Explain the acronym HIFI; if it is “high fidelity”, I suggest to just write “high fidelity”

**Response:** We have clarified as “The highly accurate long reads (HIFI)” (Line: 78).

L69: Provide reference for Flye assembler v.0.3-3

**Response:** Reference [19] is added (Line: 79).

L69: Provide reference for NCBI PGAP

**Response:** Reference [20] is added (Line: 80).

L72: Provide reference for BUSCO Database

**Response:** Reference [21] is added (Line: 81).

L74: see comment for L17

**Response:** We have revised the statement as “Colonization of *M. morganii* and mortality in honey bees” (Line: 84).

L76: When you write “adjusted”, what do you mean? What was used for dilution and/or concentration of the inoculum?

**Response:** We have clarified as “Specifically, we prepared *M. morganii* solution at OD_600_=1 and diluted with the PBS solution to 10^-3^, 10^-5^, and 10^-7^ of inoculum.” (Line: 86-87).

L76: Briefly explain what OD600=1 means: e.g., “...we adjusted the inoculum so that its optical density would be 1 at 600 nm (OD600=1)”.

**Response:** We first prepare the *M. morganii* solution at a high concentration (OD_600_=1). Then we diluted the solution for inoculation. We have clarified as “Specifically, we prepared *M. morganii* solution at OD_600_=1 and diluted with the PBS solution to 10^-3^, 10^-5^, and 10^-7^ of inoculum.” (Line: 86-87).

L76: What did you use for 10-3, 10-5 and 10-7 dilutions? I suspect it is PBS, but it must be stated.

**Response:** We have clarified as “Specifically, we prepared *M. morganii* solution at OD_600_=1 and diluted with the PBS solution to 10^-3^, 10^-5^, and 10^-7^ of inoculum.” (Line: 86-87).

L79: Specify “...to count bacterial CFU…”. Also in L83. Specify the method you have used to count CFUs (did you cultivate a known concentration of the sample? Did you manually count the colonies after 7 days?)

**Response:** We have clarified that “Dead pupae were recorded and homogenized to manually count CFU (Colony Forming Unit) using a dilution series.” (Line: 91-92).

L81: Specify “...into gnotobiotic adult honey bees”

**Response:** We have clarified that “Additionally, we injected 1μL inoculum of 10^-3^ (N=102 gnotobiotic bees), and 10^-7^ (N=116 gnotobiotic bees) into gnotobiotic adult honey bees.” (Line: 93-94).

L86: Move the [20] reference; also, as the Supplementary material only provide details regarding the BTK procedures, move here the sentence in L95-96: “...using the Bee Microbiome Toolkit [20]. Detailed procedures are provided in Supplementary Material.”

**Response:** We have revised the statement as “*M. morganii* is engineered using the Bee Microbiome Toolkit [22]. Detailed procedures are provided in the supplementary material.” (Line: 106-107).

L88: I suggest reformulating as “...can be transmitted among bees though co-housing them”.

**Response:** We have revised it as “First, we investigated whether fluorescent *M. morganii* can be transmitted among bees through co-housing them.” (Line: 99-100).

L91: Later on, it becomes clear that the “no treatment” group is the same size of the “treatment” group; nonetheless, you should make it clear here: I’d rephrase as “The second group (15 gnotobiotic bees with 5 replicates = 75 gnotobiotic bees).

**Response:** Please see the below response.

L90-94: In my opinion, this description would improve by focusing on your sample unit, and specifying the number of replicates (N=5) only once, at the end. I also suggest to rephrase for better clarity, as something like: “To this aim, we inoculated 1 µL of 10-7 M. morganii into 15 gnotobiotic bees, marking them with a colour patch placed on their thorax for identification. We co-housed these infected bees with 15 naive bees in a cup (1:1 ratio), providing five replication of the experiment (tot. 75 infected bees and 75 naive bees). As a control group, we inoculated 75 bees with 1µL PBS.

**Response:** We have revised the statement as “To this aim, we injected 1 μL of 10^-7^ inoculum into 15 gnotobiotic bees, marking them with a color patch placed on the thorax for identification. We co-housed these infected bees with 15 naive bees in a cup at 1:1 ratio with 5 replicates (N = 75 gnotobiotic infected bees and 75 naïve bees).” (Line: 100-103).

L98: Replace “vectored mites” with “infected mites”

**Response:** We have replaced “vectored mites” with “infected mites” (Line: 110).

L99-104: Here, you made a subsample of your population: you should specify how you did it (I assume it was by random selection). I also think that the overall description needs adjustments for better clarity. Here’s my suggestion: “First, we injected 25 gnotobiotic bees with the engineered M. morganii, transferring one mite to each bee’s body surface for 24 hours, providing 5 replicates of this experiment (N=125 infected bees and 125 mites): we’ll refer to this as the “injection” group. After 24 hours, we randomly selected 15 of those mites, transferring each one to a naïve bee’s body surface for 3 days (5 replicates, N=75 naive bees and 75 infected mites) as the “recipient” group. Furthermore, we injected PBS into 15 gnotobiotic bees and transferred mites to the bees’ body surface at a 1:1 ratio (5 replicates, N=75 gnotobiotic bees and 75 infected mites) as the “control” group.

**Response:** We have revised the statement as “First, we injected 25 gnotobiotic bees with the fluorescent *M. morganii*, transferring one mite to each bee’s body surface for 24 hours, providing 5 replicates of this experiment (N=125 infected bees and 125 mites): we refer to this as the injection group. At 24 hours, we homogenized the mite for CFU counting to infer the proportion of mites that acquired the fluorescent *M. morganii* (N=38 mites) from the injected bees. After 24 hours, we randomly selected 15 of those mites, transferring each one to a naïve bees’ body surface for 3 days (5 replicates, N = 75 naive bees and 75 infected mites) as the recipient group. Furthermore, we injected PBS into 15 gnotobiotic bees and transferred mites to the bees’ body surface at a 1:1 ratio (5 replicates, N=75 gnotobiotic bees and 75 mites) as the control group.” (Line: 112-120).

L107: Provide version of R and reference

**Response:** We have specified the version and citation as “The statistics were performed in R (Version 4.2.2) [23]” (Line: 123).

L107: Provide reference for each test, and move the [21] reference just after “...in the survival package”

**Response:** We have moved the reference as suggested (Line: 125). The reference for the Kruskal-Wallis test and Wilcoxon rank sum test is not required, as these tests are built into R.

L109: Provide explanation of FDR acronym, and reference

**Response:** Reference is not required for FDR because it is built into R. We have revised the statement as “Survival was analyzed using the Kaplan-Meier estimate in the survival package [24], and multiple comparisons were adjusted for False Discovery Rate (FDR).” (Line: 124-125)

L110: Provide reference for Wilcoxon test

**Response:** Reference is not required for the Wilcoxon test because it is built into R.

L114-117: Remove “Discussion” material and rephrase as something like: “We isolated 33 bacterial species (Table S1) and successfully cultivated M. morganii in LB, CBA and BHI in both regular and CO2 incubators.”
**Response:** We have revised it as suggested (Line: 130-131).

L122: Reference both Table S3 and Figure S1 (Figure S1 alone does not provide information regarding host). In Figure S2, highlight your sequence (either graphically or in the caption, e.g. by specifying “bottom Y axis”)

**Response:** We have included Figure 1 and Table S3 (Line: 136), and we have highlighted the *M. morganii* genome with color in the graph (Figure S2).

L123-124: Move the last sentence to “Discussion” (providing adequate references)

**Response:** It is deleted.

L125: As this is a “Results” sub-session, I’d stick to non-conclusive headings, e.g. “M. morganii mortality in pupae and adult bees”.

**Response:** We have revised it as “*M. morganii* induced mortality in pupae and adult bees”(Line: 138).

L126: see comment for L17

**Response:** We have revised the statement as “To investigate the impact of *M. morganii* on honey bee mortality, we injected the bacterial inoculum into the pupae and adult bees” (Line: 139-140).

L128-133: For clarity, I’d rephrase as “...~99,5%), while the injection of M. morganii resulted in a significantly high mortality at all concentrations. Specifically, the pupae survival was the lowest in the 10-3 group (34,6%, 95% CI: 25,6%~46,8, Log-rank test p<0,001)…”.

**Response:** We have revised the statement as “The pupae injected with PBS showed a high survival (98.2%, 95% CI: 96.9% ~ 99.5%), while the injection of *M. morganii* resulted in a significantly high mortality at all concentrations. Specifically, the pupae survival was the lowest in the 10^-3^ group (34.6% , 95% CI: 25.6% ~ 46.8%, Log-rank test, adjusted *P* < 0.01) compared with the 10^-5^ (61.8%, 95% CI of 55.9% ~ 62.8%; Log-rank test, adjusted *P* < 0.01) and the 10^-7^ groups (68.2%, 95% CI of 66.2%~74.8.0%; Log-rank test, adjusted *P* < 0.05, Figure 1C).” (Line: 140-145).

L139-144: As all the bees are necessarily dead after homogenation, replace “dead” with “infected”, and “live” with “non-infected”. Also in the caption for Figure 1.

**Response:** The live bees are those that survived the *M. morganii* injection. To improve clarity, we revised the statement as “To investigate whether the bee mortality was caused by the proliferation of the injected *M. morganii*, we collected dead and live individuals. We plated their homogenate to count the CFU.” (Line: 149-150). Also in the caption as “High bacterial titer was identified in dead pupae and dead bees, which is rarely found in those that survived the *M. morganii* injection.” (Line: 347-349).

L143: Replace “approximate” with “similar”

**Response:** We have replaced “an approximate” with “a similar” (Line: 154).

L144-145: Move in “Methods” (from “To investigate…” to “…in vitro”). Furthermore, I think that “symbiont” can be quite a controversial terminology, when referring to Morganella: either provide scientific support for the symbiosis, or replace with “bacterial proliferation” (See caption of Figure1 too).

**Response:** We have moved it to Methods as “To investigate the rate of *M. morganii* proliferation, we performed a time-series recording of OD_600_ in vitro.” (Line: 72-73). We have revised the caption as “The proliferation of *M. morganii* reached a stationary phase in approximately 24 hours in a series of diluted homogenates” (349-350).

L148: v. L125

**Response:** We have revised it as “The transmission of *M. morganii*” (Line: 158).

L149-151: Move to Methods

**Response:** “We selected the concentration of 10^-7^ to investigate the transmission routes of *M. morganii* among bees and mites because this concentration allows bees to survive the experiment before being killed by the infection.” has been moved to Materials and Methods (Line: 110-112).

L152-154: Move to Methods

**Response:** It has been deleted to avoid redundancy in methods.

L157: Add “At the end of the experiment, M. morganii titer…”. Also, remove “of” and open parenthesis before “1012.6 cells”

**Response:** We have revised the statement as “At the end of the experiment, *M. morganii* titer was high in the injection group (10^12.6^ cells, 95%CI: 10^11.9^~10^13.2^)” (Line: 162-163).

L164: Move “after introducing the mites to infected bees” after “Comparatively,”

**Response:** We have revised the statement as “Comparatively, after introducing the mites to the fluorescent *M. morganii* infected bees, we observed a clear fluorescence in the mites (Figure 2D).” (Line: 168-170).

L168: Rewrite as “…M. morganii, while three dead mites were uninfected”.

**Response:** We have revised the statement as “By plating the homogenates of the mites, we found the titer of *M. morganii* approached 10^4.5^ per mite (95% CI of 10^4.2^~10^4.9^, Figure 3A), after parasitizing *M. morganii* injected bees, while 3 dead mites were uninfected.” (Line: 170-172).

L172: Rewrite as “…the mites that have parasitized the injected bees…”

**Response:** We have revised the statement as “We collected the mites that had parasitized the injected bees and transferred those mites to the recipient gnotobiotic bees.” (Line: 176-177).

L174: Add the numeric results of the CFUs in the recipient group. Also, see L167-170

**Response:** We have revised the statement as “We found that *M. morganii* can colonize and persist in most bees in the recipient group, as reflected by the CFU (10^5.6^, 95% CI of 10^4.8^ ~ 10^6.3^, Figure 3B).” (Line: 177-178).

L176-177: I suggest to state this affirmatively, as “To infer whether the mortality of injected and parasitically transmitted M. morganii is similar, we compared the survival of bees infected through injection, to that of recipient bees.”

**Response:** We have revised the statement as suggested (Line: 181-182).

L178: I suggest rewriting as “At 4 dpi, we found that bees in the injection group showed a low survival (37.6%, 95%CI: 28.8-49.2%), which was even lower in the recipient group…”.

**Response:** We have revised the statement as “At 4 dpi, we found that the bees in the injection group showed a low survival (37.6%, 95% CI of 28.8% ~ 49.2%), which was even lower in the recipient group (28%, 95% CI of 16.3% ~48.2%).”(Line: 182-184).

L180: Add the numeric mortality ad 2 days after “high” (~25%).

**Response:** We have revised the statement as “The injection group showed a high mortality as early as 2 dpi (24.7%, 95% CI of 18.1% ~ 30.8%).” (Line: 184-185).

L185: I suggest rewriting as “in mites, and highlighted that this pathogenic…”

**Response:** We have revised the statement as “In this study, we isolated the first *M. morganii* in *Varroa* mites and highlighted that this pathogenic bacterium is highly virulent in honey bees.” (Line: 191-192).

L193: see comment for L17

**Response:** We have revised the statement as “thereby enhancing the case fatality rate, leading to the observed acute mortality.” (Line: 199-200).

L194: I suggest rewriting as “For example, another opportunistic pathogen of honeybees, Serratia marcescens, showed…”

**Response:** We have revised the statement as “For example, another opportunistic pathogen of honey bees, *Serratia marcescens*, caused approximately 20% mortality when infected through feeding, while injecting it leads to 100% mortality” (Line: 201-203).

L195: see comment for L17

**Response:** We have revised the statement as “For example, another opportunistic pathogen of honey bees, *Serratia marcescens*, caused approximately 20% mortality when infected through feeding, while injecting it leads to 100% mortality” (Line: 201-203).

L196: I suggest rewriting as “…mortality when infected through feeding, while injecting it led to 100% mortality [30].”

**Response:** We have revised the statement as “For example, another opportunistic pathogen of honey bees, *Serratia marcescens*, caused approximately 20% mortality when infected through feeding, while injecting it leads to 100% mortality” (Line: 201-203).

L197: I suggest replacing “data” with “study” or alike

**Response:** We have replaced “data” with “study” (Line: 203).

L201: I suggest rewriting as “…Deformed wing virus (DNW) is ubiquitously…”. Also, specify what you mean when you say that DWV titer is “low” when mites (or is it Varroa?) are absent (e.g. “relatively low in a given bee population”).

**Response:** We have revised the statement as “For example, Deformed wing virus (DWV) is ubiquitously harbored and vectored by the *Varroa* mite [39]. The titer of DWV is relatively low in honey bees when the *Varroa* mites are absent” (Line: 208-209).

L205: I suggest rewriting as “…cohousing, while it exhibited a 92.1%...”. “…mites; this is much higher than the reported 29.3%...”

**Response:** We have revised the statement as “In our study, we found that *M. morganii* couldn’t be transmitted from infected bees to naïve bees during the cohousing, while it exhibited a 92.1% transmission from infected bees to mites; this is much higher than the reported 29.3% DWV transmission from bees to mites” (Line: 211-213).

L212: See comment for L29

**Response:** We have revised the statement as “In this study, we isolated a pathogenic bacterium *M. morganii*, which is vectored by mites and causes substantial mortality in honey bees.” (Line: 218-219).

Bibliography: DOI missing for [6], [10], [12]

**Response:** We have now included the doi for [6], [10], [12].

Reviewer #2: (No Response)

Reviewer #3:

1. There is no mention what was the colony of bees that was used to obtain the mites . If it was in nature , at least the area needs to be mentioned.

**Response:** We have clarified that “We collected *V. destructor* mites from adult honey bees *A. mellifera*, using the sugar-shaking method from multiple colonies in the experimental apiary in Jiangxi Agricultural University” (Line: 64-65).

2. There is no citation for the DNA extraction method from a single colony.

**Response:** The citation is not required because Illumia provides this protocol for DNA extraction. We have clarified that “The DNA of a single colony of *M. morganii* was extracted using Sodium Dodecyl Sulfate (SDS) and sheared to 30 Kbp to be sequenced in PacBio Sequel II according to the Illumina DNA preparation instructions.” (Line: 76-77).

3. Where were on the body of the bees was the inoculum injected?

**Response:** We have clarified that “To simulate the mites’ feeding behavior, we injected the inoculum into the abdomen of pupae and gnotobiotic honey bees *A. mellifera*.” (Line: 85-86).

4. Please note that for arthropod vectors they transmit a pathogen to a host but they acquire a pathogen when feeding on an infected host. Please make changes in the entire manuscript to reflect this where necessary (eg. line 98)

**Response:** We agree and we explained in the introduction as “The naïve mites also acquire the viruses when feeding on infected bees.” (Line: 40-41), and Results section as “Secondly, we investigated the transmission from infected bees to naïve *Varroa* mites” (Line: 167), and “Thirdly, we investigated the transmission of *M. morganii* from infected *Varroa* mites to naïve bees.” (Line: 175).

5. Line 155 and Line 176 grammatically incorrect, change.

**Response:** We have replaced “investigate” by “investigated” (Line: 167). We have revised the sentence as “To infer whether the mortality of injected and parasitically transmitted *M. morganii* is similar, we compared the survival of bees infected through injection, to that of recipient bees.” (Line: 181-182).

6. Figure S1 - Legend - A new strain versus species was identified. Cannot be a new species if you refer to it as M. morganii.

**Response:** We have revised the legend as “The one isolated from the *Varroa* mite shows an average identity of 92%. The genome assembled in this study is highlighted.” (Figure S1).
